# Concomitant Occurrence of Hepatitis A and a Pyogenic Liver Abscess: A Pediatric Case

**DOI:** 10.7759/cureus.60288

**Published:** 2024-05-14

**Authors:** Ekta Kumari, Zohaa Shahid, Fatima Shakeel

**Affiliations:** 1 Pediatric Infectious Diseases, Liaquat National Hospital and Medical College, Karachi, PAK

**Keywords:** dual infection, liver abscess, pyogenic liver abscess (pla), hepatitis a virus (hav), amoebic liver abscess, hepatitis a serology, hepatomegaly, pleural effusion, complicated liver abscess, hepatitis

## Abstract

Pyogenic liver abscess (PLA) and hepatitis A are common in developing countries. As there is an overlap of clinical features, a diagnosis of dual infection can be missed. Here, we present the case of a five-year-old male who presented with abdominal pain, fever, and jaundice diagnosed as a complicated liver abscess with concurrent hepatitis A. To our knowledge, this is the first case where a PLA co-existed with hepatitis A. Simultaneous infection should be considered when a patient with liver abscess presents with jaundice, especially in areas where both diseases are endemic. Early diagnosis of both is crucial as PLA is a potentially fatal disease and co-infection with hepatitis A may worsen clinical outcomes.

## Introduction

Due to financial constraints and limited resources, dual infections are likely to be missed in low socioeconomic areas. Given the similar presentation of both liver abscesses and hepatitis A, it is not uncommon to overlook the other.

Pyogenic liver abscess (PLA) is common in developing countries [1]. Patients with immunodeficiency, anemia, and low socioeconomic status are at a higher risk [2]. The typical symptoms include fever, abdominal pain, and reduced appetite [2]. Diagnosis is confirmed by abdominal imaging, usually via ultrasound or CT scan, which typically shows intrahepatic fluid-filled lesions with surrounding edema. Treatment options include percutaneous drainage of the abscess followed by a four to six-week course of antibiotics [3]. PLA is a potentially lethal condition making earlier diagnosis and treatment essential to prevent complications that include sepsis, pneumonia, and rupture of abscess in the peritoneum or thorax.

Hepatitis A is a common infection worldwide with a relatively higher prevalence in developing countries [4]. Although it can present with symptoms ranging from mild to severe, interestingly, it is usually asymptomatic in children [5]. Clinical features of hepatitis A and PLA overlap and present with nausea, vomiting, anorexia, abdominal tenderness, and hepatomegaly; however, jaundice is predominantly associated with hepatitis A [4]. Diagnosis of hepatitis A requires detection of immunoglobulin M antibody to hepatitis A virus (HAV) [5].

## Case presentation

A five-year-old male presented to the emergency department complaining of progressive abdominal pain, distention, and jaundice for the past seven days with the background of ongoing fever for a month. Fever was initially reported as low grade that progressed to high grade over the last week. The patient had been taking over-the-counter antipyretics to treat the fever. Jaundice was abrupt in onset, worsening with time, and associated with pale stools and dark urine. Abdominal pain was reported in the right upper quadrant and aching in character. The patient reported no other gastrointestinal symptoms. A review of systems revealed decreased appetite, generalized malaise, and undocumented weight loss in the last one to two months. His history was otherwise unremarkable.

On examination, he appeared sick, icteric, lethargic, and pale. He was oriented to time, place, and person. Vital signs on presentation revealed a heart rate of 107 beats/minute, respiratory rate of 30 breaths/minute, temperature of 37.8°C, blood pressure of 109/69 mmHg (90th percentile) oxygen saturation of 98%, weight of 18 kg (50th percentile), and height of 116 cm (90th percentile). Abdominal examination revealed a distended abdomen, tenderness in upper quadrants with abdominal guarding secondary to pain, and a palpable liver 4 cm below the right costal margin. The complete measurement of the liver was limited due to pain on examination. Shifting dullness was present without a fluid thrill. Gut sounds were present and normoactive. No other significant examination findings were noted.

On admission, blood investigations (Table 1) were significant for anemia, leukocytosis with a left shift, elevated inflammatory markers, elevated conjugated bilirubin, low albumin levels, and deranged liver function test. Investigations to ascertain the underlying etiology (Table 2) revealed hepatitis A serology (IgM) positive. Furthermore, ultrasonography of the liver showed an enlarged liver measuring 14.5 cm with a complex ill-defined lesion measuring 11 × 11.6 cm in segment VI of the right lobe of the liver with posterior acoustic enhancement consistent with PLA.

**Table 1:** Laboratory investigations. PCV = packed cell volume; TLC = total leucocyte count; CRP = C-reactive protein; ALP = alkaline phosphatase; GGT = gamma-glutamyltransferase; AST = aspartate aminotransferase; ALT = alanine aminotransferase; PT = prothrombin time; aPTT = activated partial thromboplastin time; INR = international normalized ratio

Investigations	Day 1	Day 3	Day 8	Day 13	Normal values
Hemoglobin	8.4	8.7	5.9	11.6	13.5–17.5 g/dL
PCV	27	28.4	20	36	35.5–48.6%
Platelets	279	244	192	282	150,000–400,000/mm^3^
TLC	31	23.9	14.4	6.9	4–11 × 10^3^/mL
Neutrophils	73	75	80	82.5	54–62%
Lymphocytes	13	13	15	14.6	25–33%
CRP	28.9	1.08	1.32	-	<0.3 mg/dL
Total bilirubin	10.6	4.7	-	-	0.1–1.0 mg/dL
Direct bilirubin	9.6	4.2	-	-	0.0–0.3 mg/dL
Indirect bilirubin	0.9	0.5	-	-	0.2–0.8 mg/dL
ALP	630	692	-	-	25–100 mg/dL
GGT	167	154	-	-	0–30 IU/L
AST	382	142	-	-	12–38 U/L
ALT	202	54	-	-	10–40 U/L
Albumin	2.08	3.06	-	-	3.5–5.5 g/dl
PT	16	-	-	-	11–15 seconds
aPTT	29	-	-	-	25–40 seconds
INR	1.6	-	-	-	2.0–3.0

**Table 2:** Workup to determine the underlying etiology. MTB PCR = multidrug-resistant tuberculosis polymerase chain reaction; HAV = hepatitis A virus; IgM = immunoglobulin M; HEV = hepatitis E virus; *P. falciparum* = *Plasmodium falciparum*; NS1 = non-structural protein 1; HBsAg = hepatitis B surface antigen; HCV = hepatitis C virus

Investigations	Results
Pus culture	Negative
MTB PCR	Negative
Anti HAV (IgM)	Reactive
Anti HEV (IgM)	Negative
Blood culture	Negative
Stool panel with ova and parasite	Negative
*P. falciparum* serology	Negative
Non-*P. falciparum* serology	Negative
Dengue NS1	Negative
HBsAg	Negative
HCV RNA	Negative

**Figure 1:**
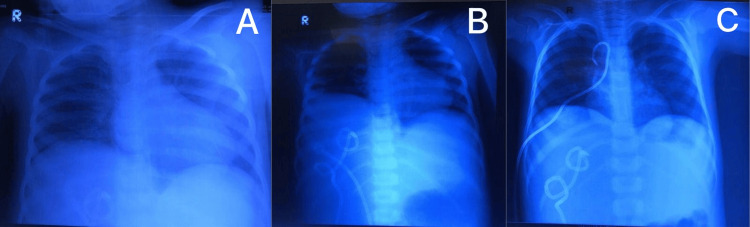
Course of development of pleural effusion. A: Day one of admission with no signs of effusion. B: Day four of admission with pleural effusion. A pigtail placed for a liver abscess on day two can be seen. C: After the placement of a pigtail for pleural effusion.

The patient was managed in a ward setup. Intravenous fluids were administered, and intravenous antibiotics (meropenem + vancomycin + metronidazole) were commenced concurrently with albumin replacement. An ultrasound-guided pigtail was placed for abscess drainage by an interventional radiologist on day two of admission. Initially, on admission, the chest X-ray was unremarkable. However, he developed sudden shortness of breath on the fourth day of admission for which a chest X-ray was repeated revealing a right-sided pleural effusion (Figure 1). This was managed by placing an ultrasound-guided pigtail inside the pleural cavity and antibiotics were continued. On the eighth day of admission, a precipitous drop in hemoglobin and platelets was discovered, the cause of which was never elucidated. However, he was transfused with packed red blood cells. Gradually, his condition improved, oral intake increased and physical activity enhanced; hence, he was discharged on the 17th day of admission on oral antibiotics. Pigtails for liver abscess and pulmonary effusion were removed on the seventh and tenth post-discharge days, respectively. He was followed weekly in the outpatient department and showed good progress during the follow-ups regarding both chest and abdominal symptoms.

## Discussion

We present the case of a five-year-old male patient who experienced a prolonged infectious illness of hepatic origin with acute worsening of symptoms. After workup for etiology, it became apparent that the patient had PLA with concomitant hepatitis A infection. To our knowledge, this is the only case reported where PLA existed simultaneously with hepatitis A infection.

PLA is uncommon in the pediatric population; however, it is the most common type of liver abscess seen in pediatric patients [1,3]. Clinical findings of PLA are similar to those of hepatitis A and include enlarged liver, vomiting, nausea, anorexia, abdominal pain, and tenderness. PLA commonly presents with normal body temperature and a negative blood culture, as highlighted in our case [6]. These features have the potential to cause oversight when evaluating infectious etiology and require a thorough workup.

PLA is more likely to present in patients with older age, predisposing liver disease, or immunodeficiency [2,7,8]. This case differed in that the patient was previously healthy and there was no history or features suggestive of immunodeficiency. Although there is some evidence to suggest hepatitis A [9,10] predisposes to other infections, research on this topic is limited, and the timeline of this case presents uncertainty as to whether PLA preceded hepatitis A or vice versa.

A finding that warrants attention in this case is the elevated bilirubin level. Though PLA is more likely than amoebic abscess to present with elevated bilirubin levels [11], it is an uncommon finding in PLA [12]. The probable explanation for the elevated bilirubin is the concurrent hepatitis A infection [5]. Hepatitis A also explains the hepatocellular pattern of liver injury seen in this case, characterized by high serum levels of the enzymes alanine aminotransferase and aspartate aminotransferase, as well as serum levels of alkaline phosphatase and gamma-glutamyltransferase.

Previously described cases where hepatitis A co-presented with liver abscess are limited to the amoebic variety. These were found in individuals who had traveled to a developing country [13] or an endemic region [14]. Given the higher prevalence of both PLA [15] and hepatitis A [16] in developing countries, it seems more probable that concurrent infection was due to chance. However, the acute worsening of symptoms with which the patient presented as well as the complicated disease course suggest that dual infection may be a more detrimental combination.

Our case is limited by a few variables. Due to financial constraints, the serology of hepatitis A was not monitored after treatment initiation. More data on the serology could have facilitated a better understanding of the timeline of hepatitis A infection. Furthermore, the setting in a center with limited resources posed difficulties regarding the collection of complete data. As a result, data on liver function tests for day eight onward are unavailable which limits a thorough workup and interpretation of the case.

## Conclusions

Dual infections of hepatic origin have rarely been reported. Endemic regions where these diseases are common are limited in their resources for diagnosis and treatment. Dual infections may easily be missed and possibly result in worse patient outcomes due to increased chances of complications.

Challenges with establishing a correct diagnosis exist, especially when both diseases originate from the liver. Physicians may not order workups for other diseases when a diagnosis of one disease has already been established. This is especially true in a setting where financial constraints exist. It is, therefore, crucial to be able to differentiate the clinical and laboratory parameters of PLA and hepatitis A which present similarly with minor variations.

To our knowledge, this is the first reported case where PLA and hepatitis A occurred simultaneously. Additional cases will help further establish a possible pathophysiological link, differentiate clinical features in cases of dual infection, plan appropriate treatment regimens, and assist in predicting clinical outcomes.
